# Range-shifter effects on the stray field in proton therapy measured with the variance–covariance method

**DOI:** 10.3389/fonc.2022.882230

**Published:** 2022-08-02

**Authors:** Linda Eliasson, Jan Lillhök, Torbjörn Bäck, Robert Billnert-Maróti, Alexandru Dasu, Malgorzata Liszka

**Affiliations:** ^1^ Department of Physics, KTH, Stockholm, Sweden; ^2^ The Swedish Radiation Safety Authority, Solna, Sweden; ^3^ Medical Radiation Sciences, Department of Immunology, Genetics and Pathology, Uppsala University, Uppsala, Sweden; ^4^ The Skandion Clinic, Uppsala, Sweden

**Keywords:** LET, TEPC, variance-covariance method, dose-mean lineal energy, out-of-field dose, dose equivalent, proton therapy, pencil beam

## Abstract

Measurements in the stray radiation field from a proton therapy pencil beam at energies 70 and 146 MeV were performed using microdosimetric tissue-equivalent proportional counters (TEPCs). The detector volumes were filled with a propane-based tissue-equivalent gas at low pressure simulating a mean chord length of 2 μm in tissue. Investigations were performed with and without a beam range shifter, and with different air gaps between the range shifter and a solid water phantom. The absorbed dose, the dose-mean lineal energy, and the dose equivalent were determined for different detector positions using the variance–covariance method. The influence from beam energy, detector- and range-shifter positions on absorbed dose, LET, and dose equivalent were investigated. Monte Carlo simulations of the fluence, detector response, and absorbed dose contribution from different particles were performed with MCNP 6.2. The simulated dose response for protons, neutrons, and photons were compared with, and showed good agreement with, previously published experimental data. The simulations also showed that the TEPC absorbed dose agrees well with the ambient absorbed dose for neutron energies above 20 MeV. The results illustrate that changes in both dose and LET variations in the stray radiation field can be identified from TEPC measurements using the variance–covariance method. The results are in line with the changes seen in the simulated relative dose contributions from different particles associated with different proton energies and range-shifter settings. It is shown that the proton contribution scattered directly from the range shifter dominates in some situations, and although the LET of the radiation is decreased, the ambient dose equivalent is increased up to a factor of 3.

## 1 Introduction

Compared to conventional photon therapy, proton therapy has the potential of reducing exposure and radiation risks outside the target volume (1). Nevertheless, there is still a concern that stray radiation can increase secondary cancer risks. In an ongoing task within EURADOS working group 9, the relation between the most critical treatment parameters and the out-of-field neutron doses is therefore investigated. The outcome will hopefully be a first step toward a tool for medical physicists to estimate the neutron doses directly from the treatment parameters. Simulations and results from experimental campaigns are further described in Van Hoey et al. (2).

In the EURADOS WG9 campaign, the quantity used for comparison was the neutron ambient dose equivalent, $H_{\text{n}}^{*}\left(10\right)$ (3). During the measurement campaign at the Skandion Clinic in 2019, the Swedish Radiation Safety Authority contributed with three instruments, a Berthold LB6411 neutron monitor and two tissue-equivalent proportional counters (TEPCs), further called the Sievert detectors. TEPCs can detect and separate both high- and low-LET components, which makes them suitable for mixed radiation fields.

With varying different treatment settings in the experiment described by Van Hoey et al. (2), $H_{\text{n}}^{*}\left(10\right)$ was measured with different detectors in various positions around a solid water phantom. In one position, a significant increase in absorbed dose was measured with the Sievert detector when the range shifter was inserted. This increase was not supported by simulations or measurements performed by neutron monitors in the vicinity of the same position. A hypothesis was that scattered protons from the range shifter contributed to the absorbed dose in positions that were less shielded by the phantom.

Prior to the campaign described by Van Hoey et al. (2), several measurements as well as simulation comparisons of out-of-field doses have been conducted. It is, e.g., well known that the stray neutron fields are characterised by a thermal and high-energy component (4, 5). Range shifters and their effect on the stray neutron field have also been studied, and alternative methods to scan shallow tumours have been reported to decrease the high-LET contribution to the dose (6). However, proton scattering from the range shifter is rarely considered.

The ambient dose equivalent, *H* ^*^(10), is defined in terms of the dose equivalent *H* = *D* · *Q* (*L*) at 10-mm depth in the ICRU sphere in an expanded and aligned radiation field (7, 8). Here *D* is the absorbed dose in tissue and *Q* (*L*) is a quality factor that depends on the unrestricted linear energy transfer (*LET*) of charged particles in water. The linear energy transfer can be estimated by the lineal energy, y, measured with TEPCs simulating a tissue volume in the micrometre range (9). The distribution of y-values hence corresponds to the LET distribution of the radiation field. A change in this distribution, or, e.g., the dose-mean lineal energy ${\bar{y}}_{D},$ reflects a change in the LET of the radiation field.

In addition to microdosimetric single-event measurements, the variance–covariance method (10) has been used for radiation protection applications in mixed fields. Lillhök et al. measured differences in the stray radiation fields between photon and proton therapy (11). Several cosmic radiation measurements have been performed, where the variance and variance–covariance methods have been compared with other methods (12, 13) and with several instruments (14). The method has also successfully been used in mixed workplace fields with photons and neutrons and strongly pulsed stray radiation fields from accelerators (15, 16). Single-event measurements are limited in high-intensity fields due to pile-up which can be the case, e.g., in a therapeutic beam. As described in Lillhök et al. (13), TEPCs used for both single- and multi-event measurements showed good agreement in the mixed field onboard an aircraft and showed that it can be used as a complementary method in mixed fields.

In the investigation presented in this article, measurements complementary to the EURADOS 2019 campaign (2) were performed at the Skandion Clinic, aiming to study the dose contribution and LET of the stray radiation component from the range shifter using two Sievert detectors, which as previously described are multi-event TEPCs. The absorbed dose and dose-mean lineal energy were measured using the variance–covariance method. A phantom was placed in the same position as in the 2019 campaign and irradiated with two different proton beam energies for a variety of different range-shifter settings. Measurements were also performed without a phantom to quantify the range-shifter component directly. The detector absorbed dose responses for neutrons, protons, photons, and electrons were simulated using MCNP 6.2. These response functions were used together with simulated fluence distributions at the detector positions to evaluate the relative absorbed dose contributions from different radiation field components.

## 2 Method

### 2.1 Experimental method and equipment

The Sievert detectors are made of A-150 plastic, contained in a 2-mm-thick aluminum container and filled with propane-based tissue-equivalent gas (17) to a pressure of 1.37 kPa, corresponding to a simulated tissue volume with a mean chord length of 1.88 μm. The instruments are cylindrical with diameters and heights equal to 11.54 cm and with an A150-plastic wall of thickness 5 mm. The electric charge generated in each detector is measured using a 1-nF feedback capacitor, where the voltage over the capacitor is measured 10 times per second with a 24-bit analogue-to-digital converter (ADS 1210U). One bit is used for polarity and the other 23 bits for dividing the maximum capacitor voltage of 5 V into steps of 0.6 μV. The electronic noise is dominated by the 0.6-μV (rms) contribution from the analogue-to-digital-converter (18). The absorbed dose to the detector gas during the integrated time is determined by


(1) $$D_{det}=\frac{qW}{Mm_{det}},$$

where *q* is the electric charge collected during the integration time, *W* is the mean energy expended to create an ion pair (in energy per charge), *M* is the gas multiplication factor, and *m_det_* is the detector gas mass. The dose-mean lineal energy is calculated using the variance–covariance method,


(2) $${\bar{y}}_{D}=\frac{m_{det}\left(V_{rel}-C_{rel}\right)}{{\bar{l}}_{\mu }}{\bar{D}}_{det},$$

where *V_rel_* is the relative variance in the absorbed dose during the repeated charge, *C_rel_* is the relative covariance between two detectors experiencing the same field, ${\bar{l}}_{\mu }$ is the mean chord length for the simulated tissue, and ${\bar{D}}_{det}$ is the average dose over repeated integration times (10). In a time-varying radiation field, a covariance correction is usually determined using a second detector. However, in some situations the radiation field variations at the two detector positions are not necessarily synchronised in time. In such cases, a covariance correction can be obtained from the consecutive charge integrations. This method is further described in Eliasson et al. (article in progress) and was used in the measurements presented here.

In a mixed field, where several components contribute, the measured total ${\bar{y}}_{D}$ value is given by a combination of the relative dose contributions and their dose-mean lineal energies. As an example, the case with three components can be written as


(3) $${\bar{y}}_{D}=d_{\gamma }{\bar{y}}_{D,\gamma }+d_{n}{\bar{y}}_{D,n}+d_{p}{\bar{y}}_{D,p},$$

where *d_i_* is the relative dose contribution for photons, neutrons, and protons, respectively. From the equation, it is clear that a change in any of the component contributions will be reflected as a change in the measured ${\bar{y}}_{D}$ value. As previously mentioned, the dose-mean lineal energies for the radiation components are energy dependent and can be estimated by using their simulated fluence distributions for the stray field and a known response function determined in monoenergetic beams (18, 19). The simulated response function and comparisons with measurements are described in the following sections. While the ${\bar{y}}_{D,\gamma }$ value depends strongly on the neutron energy, it is in stray fields from proton therapy dominated by high-energy neutrons, giving a ${\bar{y}}_{D,n}$ value typically around 100 keV/μm (1, 2), while the ${\bar{y}}_{D\gamma }$ value is typically around 1.5 keV/μm (2, 11). For the proton component, the ${\bar{y}}_{D,p}$ value for a 1-μm-diameter spherical object in water is approximately in the range 2–6 keV/μm for proton energies between 10 and 100 MeV but increases with decreasing energies. At 1 MeV, the ${\bar{y}}_{D,p}$ value is approximately 40 keV/μm (20). Moreover, Kyllönen et al. measured dose-mean lineal energies in proton beams between 68 and 174 MeV with the Sievert detectors and reported ${\bar{y}}_{D}$ values between 6.2 and 7.3 keV/μm (21).

Just as for the resulting ${\bar{y}}_{D}$ value, the *W* value depends on the radiation components. However, the *W* values of photons, neutrons, and protons [26.8, 31, and 28.2 eV (22)] do not differ as dramatically from each other as the dose-mean lineal energies. Previous simulations of the stray field from a proton beam reported that the relative dose distribution from neutrons varied from approximately 54% to 95%, giving a W value that varied between 28.9 and 30.7 eV (11). The addition of a proton component does not change the mean value significantly, so the same mean value as reported by Lillhök et al. (11) was used for all irradiations in this article as well, and the variations were handled in the uncertainty estimation.

The *H* ^*^(10) values were estimated from a measured dose equivalent, *H* ^*^, using a first-order approximation for the quality factor,


(4) $$H^{*}=DQ_{D}=D\;(a+b{\bar{y}}_{D}),$$

where *a* = 0.73 and *b* = 0.17 μm/keV. (19). For this article, only total *H* ^*^ values were determined, but it is possible to estimate the *H* ^*^ values for the high- and low-LET components by using the relative dose contribution for each radiation component and the respective ${\bar{y}}_{D}$ value in Equation 3.

No calibration factors relating the detector readings to *D* ^*^(10) or *H* ^*^(10) directly are used. The absorbed dose (Equation 1) and dose-mean lineal energy (Equation 2) in the tissue-equivalent detector material are derived from traceable measures of air pressure and electric charge, where the physical detector volume with uncertainties is assumed to be representative of the true charge collecting volume. For the dose-equivalent measurements based on Equation 4, the constants *a* and *b* have been optimised for a neutron beam with a broad energy spectrum (19).

### 2.2 Experimental setup

The experiment was conducted at the Skandion clinic, which has been in operation since 2015 and is the first centre for proton beam therapy in the Nordic countries and the only centre situated in Sweden. By optimising the dose delivery to the target volume, the clinic can treat patients with tumours close to vital organs, reducing both the risk for secondary cancer and long-term side effects ^^1^^.

**Figure 1:**
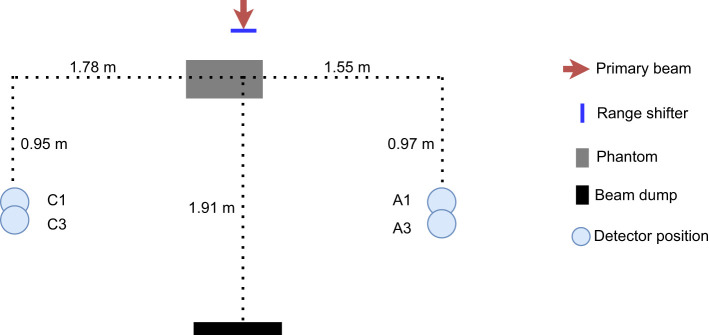
Schematic drawing of the experimental setup, to scale. Two detectors were used during the irradiation: one was placed on the C side, situated out toward the treatment room, and the other was situated at position A, close to the gantry wall, The phantom was positioned with its centre 15 cm from the beam line.

**Figure 2:**
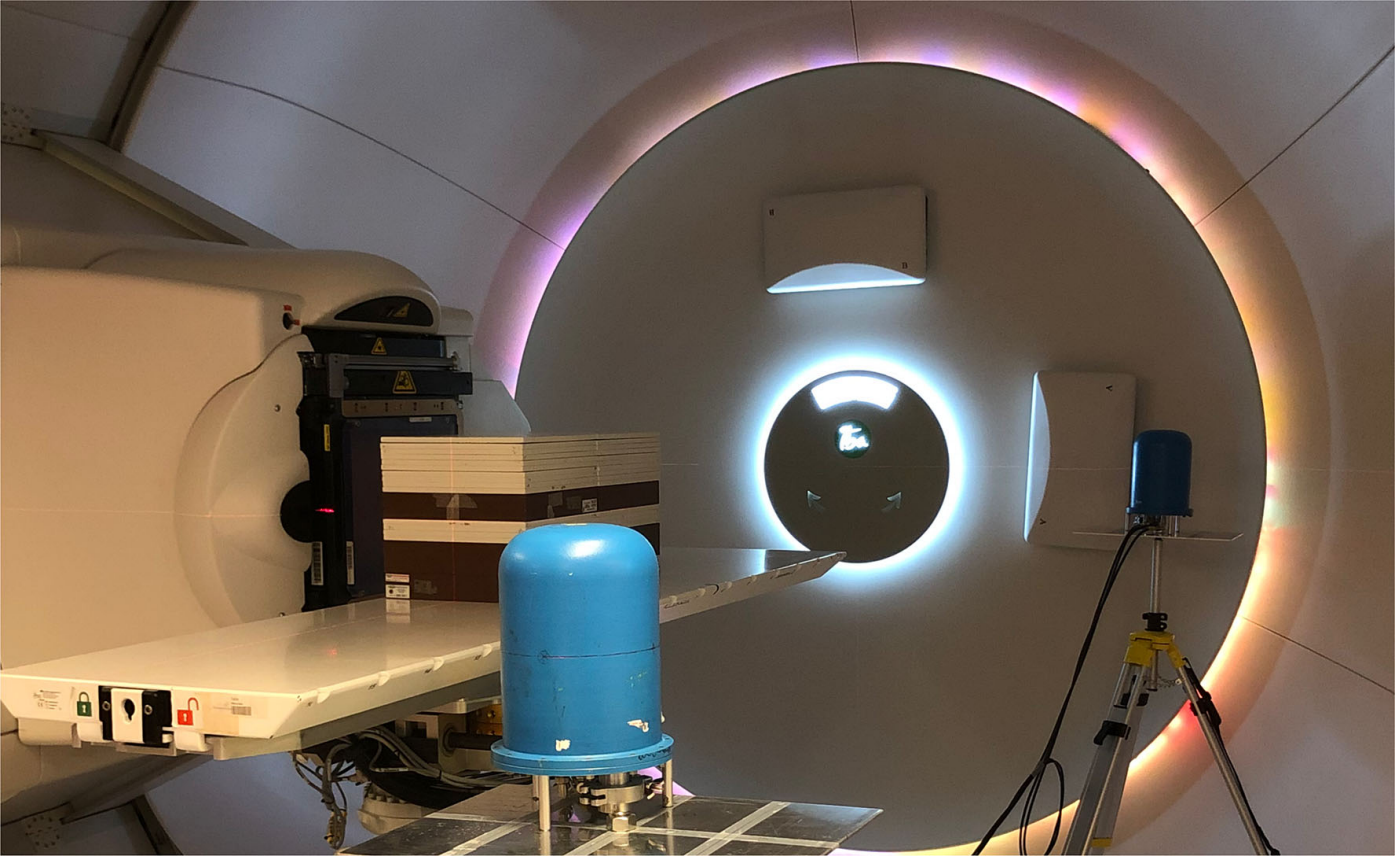
The experimental setup, with the two TEPCs in positions A and C. The C position is situated close to the gantry wall, while the A position in the foreground is facing the open treatment room.

The proton beam at the facility is an IBA pencil beam and delivers protons with energies between 60 and 226 MeV (23–25). A schematic illustration to scale and a photo of the setup are seen in **Figures 1**, **2**. To better investigate the differences between range-shifter settings, only single-spot measurements were conducted, using a pristine beam. The detector positions A1 and C1 were similar to positions A and C in the 2019 campaign (2), while A3 and C3 were 10 cm further down the beam line. During the measurements, two detectors were used, where one detector was placed in any of the positions on the A side (A1 or A3) and the other was placed in the corresponding position on the C side (C1 or C3). The proton current was set to the same value, 0.6 nA, for all irradiations. A solid water (polystyrene) phantom of density 1.03 g/cm^3^, height and width equal to 30 cm, and length 60 cm was placed with its centre shifted 15 cm from the beam line. The range shifter is made of Lexan (polycarbonate) with a thickness of 3.11 cm and density of 1.20 g/cm^3^. Measurements were performed without the range shifter and with the range shifter at different air gaps (AG) from the phantom wall. For irradiations performed without the phantom, a proton beam dump was positioned at the far wall. The beam dump is made of PMMA with density 1.18 g/cm^3^, height 32 cm, thickness 6.5 cm, and length 40 cm. A total of32 irradiations were performed with two different detector positions, two different proton beam energies, and a number of different range-shifter settings.

### 2.3 Simulations

The absorbed dose response of the detector and the particle fluence distributions in the different measurement positions were simulated with MCNP version 6.2 (26) in order to support and extend the analysis of the measurement results. The response function for protons, neutrons, photons, and electrons were combined with the respective fluence distributions to calculate the relative absorbed dose contribution from each particle type.

#### 2.3.1 Absorbed dose response of the Sievert detector

**Figure 3:**
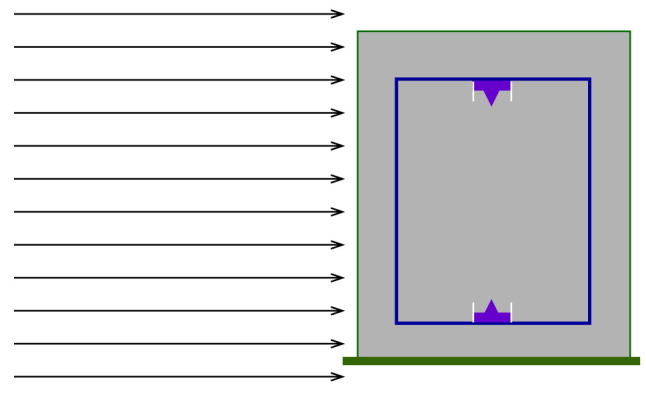
An illustration of the simulation geometry for the detector dose response simulations in MCNP 6.2. The cylindrical detector is confined in an aluminium container and exposed to monoenergetic beams.

The absorbed dose response was simulated using a detector model in vacuum as shown in **Figure 3**, exposed to a parallel beam of monoenergetic particles with a beam diameter of 40 cm. The detector was filled with propane-based tissue-equivalent gas to a density of 26.12 μg/cm^3^, corresponding to a mean chord length of 2 μm tissue. The detector geometry was modelled as described in Section 2.1.

Simulations were carried out for 10^-10^–10^4^-MeV neutrons, 1–10^4^-MeV protons, 10^-2^–10^4^-MeV photons, and 10^-2^–10^4^-MeV electrons, using LA150 data libraries. Primary and secondary particles (photons, electrons, neutrons, protons, alpha-particles, deuterium, tritium ^3^He, and heavy ions) were transported in all volumes. The absorbed dose in the cylindrical detector gas volume was scored using the +F6 tally for total heating. The +F6 tally scores energy depositions from all particles and not only a specific particle type. A linear energy binning was used for all particles with 10 bins per decade for neutrons and 20 bins per decade for protons, photons, and electrons. The total heating, i.e., absorbed dose, was normalised to the particle fluence.

#### 2.3.2 Fluence distribution at the measurement positions

The fluence simulations were performed for a simple geometry only including a range shifter, a phantom, and a beam dump. The range shifter was modelled with dimensions 3.11 cm × 15 cm × 15 cm, and the solid water phantom with dimensions as described in Section 2.2. Monoenergetic protons of energies 70 and 146 MeV in a circular and parallel beam of diameter 1 cm was transported through air to the range shifter positioned with different air gaps to the phantom. In order to quantify the effect of the range shifter, simulations were also performed with only the range shifter and no phantom, as well as only phantom with no range shifter. To further test the robustness of the relatively simple geometry, additional simulations were performed using a more comprehensive model with walls, floors, and the surrounding gantry structure (27).

The fluence distributions of neutrons, protons, photons, and electrons were first scored with tally F4 in spherical air volumes located in the same positions as the detectors in the measurements. Standard MCNP libraries were used, and photons, electrons, neutrons, protons, and alpha-particles were transported in all volumes. A linear energy binning was used for all particles with 10 bins per decade for neutrons and 20 bins per decade for protons, photons, and electrons. The fluence was normalised to the number of initial protons.

#### 2.3.3 The simulated absorbed dose

The fluence distributions and the detector response functions described above were used to estimate the relative dose contribution from neutrons, protons, photons, and electrons. The absorbed dose in the detector for particle type *i* is given by


(5) $$D_{i,TEPC}=\sum_{k}d_{i,TEPC}\left(E_{k}\right){\text{Φ}}_{i}\left(E_{k}\right)\text{Δ}E_{k},$$

where *d_i_*,*_TEPC_* is the absorbed dose response per fluence of particle type *i* with energy *E_k_* per energy bin, Φ(*E_k_*) is the fluence of particle type *i* with energy *E_k_* per energy bin, and Δ*E_k_* is the bin width with the average energy *E_k_* in the fluence distribution. *D_i_*,*_TEPC_* is therefore summed over all energy bins and gives the total absorbed dose for one particle type *i*.

### 2.4 Uncertainties

**Figure 4:**
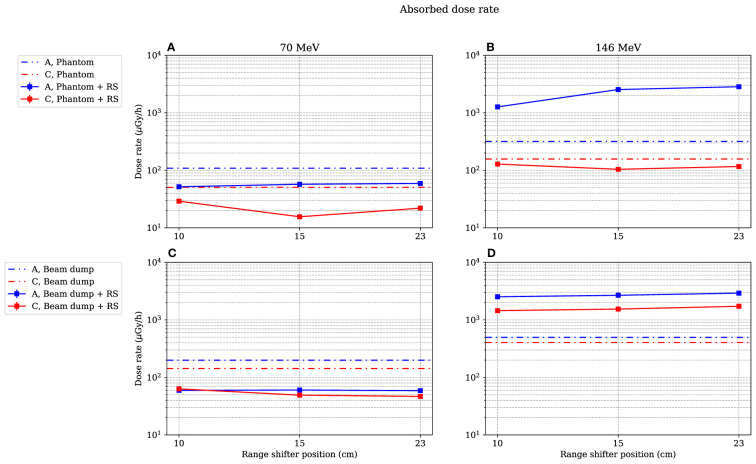
Absorbed dose rate for both 70-MeV (left figures) and 146-MeV (right figures) proton beam irradiations. The range-shifter positions correspond to the air gaps when the phantom was present. In **(A, B)**, the phantom was irradiated, while in **(C, D)**, no phantom was present and the beam was instead irradiating the proton beam dump situated by the far wall. The lines between data points are used as guide for the eye. The dashed–dotted lines show the levels without a range shifter, i.e., scattered from the phantom or from the beam dump. As mentioned in Section 2.4, the values are illustrated with statistical uncertainties only with k = 1.

**Figure 5:**
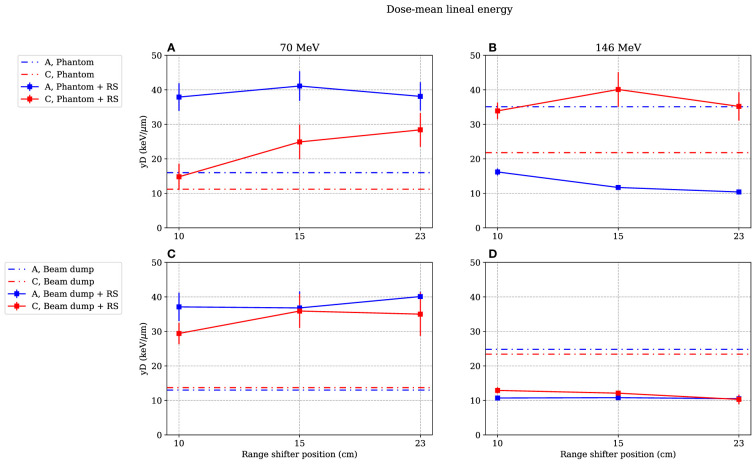
Dose-mean lineal energies as functions of range-shifter positions when irradiating the phantom **(A, B)** and the proton beam dump **(C, D)**. The range-shifter positions correspond to the air gaps when the phantom was present. Irradiating with 70-MeV protons, the range shifter leads to a higher ${\bar{y}}_{D}$ value at both positions A and C, while with 146 MeV, the ${\bar{y}}_{D}$ value measured at position A is lower and decreases with increased range-shifter position with the presence of a phantom **(B)**. When irradiating the beam dump with 146-MeV protons, the ${\bar{y}}_{D}$ values decrease both at position A and position C **(D)** The lines between data points are used as guide for the eye. The dashed–dotted lines show the levels without range shifter, i.e., scattered from the phantom or from the beam dump. As mentioned in Section 3.4, the values are illustrated with statistical uncertainties only with k = 1.

The statistical uncertainties were estimated for the absorbed dose rate and for the dose equivalent using conventional error propagation. For the dose-mean lineal energy, the statistical uncertainty was obtained by splitting each data set into smaller subsets and calculating the standard deviation of the mean. In the results presented in **Figures 4**, **5** and **9**, **10**, and both tables below, only the statistical uncertainties are included, with coverage factor k = 1. The coverage factor was chosen to harmonise with the uncertainties presented in Van Hoey et al. (2). In addition, uncertainties of the gas pressure, detector volume and diameter, electric charge, W-value, gas multiplication, *H* ^*^(10) response, and accelerator reference data need to be accounted for. The gas pressure has an uncertainty of 0.17%, estimated from calibrations of the pressure gauge at the Swedish National Metrology Laboratory for Pressure and Vacuum (RISE). The uncertainty in the generated electric charge is estimated to be 0.5% from cross-calibrations with a reference electrometer at the Swedish National Metrology Laboratory for Ionising Radiation. The uncertainties in the detector diameter and height are estimated to be 0.25% and 1.5%, respectively. Uncertainties in the effective charge collecting volume of the detector have not been taken into account, and the uncertainty in the volume was calculated directly from the dimensions. For the W value, the uncertainty was estimated to be 4%, which is the reported uncertainty for *W_p_* (22). The other *W* values (*W_n_* and *W_γ_*) are reported with smaller uncertainties (22). The gas multiplication uncertainty (0.8%) was estimated from measurements in a calibrated ^137^Cs field prior to and after the Skandion measurements. These above uncertainties give approximately 4% uncertainty contribution to add to the absorbed doses, the dose-mean lineal energies, and the dose equivalents. Since the Sievert instrument measures the absolute dose and variance between a series of 0.1-s charge collections, it is important that the proton current for each energy setting is stable, while the absolute value of the proton current is less relevant. The dosimetry uncertainty budget from the Skandion Clinic ensures that the relative dose variation is within 2% (11). This was confirmed by the clinic’s monitoring ionisation chambers.

The uncertainty in the H*(10) response of the detectors has not been included. This contribution varies with the relative contributions of the radiation components and has been reported to be about 15%–25% in typical mixed field applications (18).

The simulations are reported with statistical uncertainties with the coverage factor k = 1. Uncertainty contributions from, e.g., interaction cross sections and deviations between the simulated and real geometry, have not been taken into account.

## 3 Results

The differences in measured absorbed doses and dose-mean lineal energies between positions 1 and 3 were small, so only the results from positions A1 and C1 are reported here. The positions are onward referred to as position A and position C. The absorbed dose, *D*, the dose-mean lineal energy, ${\bar{y}}_{D}$ , and the dose equivalent, *H* ^*^, as functions of different range-shifter positions are presented for 70- and 146-MeV primary proton beams. The range-shifter settings 10, 15, and 23 cm correspond to air gaps of 10, 15, and 23 cm when the phantom is present and are in the figures referred to as AG. The experimental results are also presented in **Tables 1**, **2**.

**Table 1:** Measured absorbed dose rates and ${\bar{y}}_{D}$ values for different irradiation settings.

Proton energy (MeV)	Irradiated target	AG (cm)	Position	Dose rate (μGy/h)	${\bar{y}}_{D}$ (μGy/h)
70	Phantom	NoRS	A	109.1 ± 0.2	16.0 ± 2.3
70	Phantom	10	A	51.8 ± 0.2	37.9 ± 4.0
70	Phantom	15	A	57.3 ± 0.2	41.1 ± 4.3
70	Phantom	23	A	59.1 ± 0.2	38.1 ± 4.1
70	Beam dump	NoRS	A	199.1 ± 0.3	13.0 ± 1.0
70	Beam dump	10	A	59.5 ± 0.2	37.1 ± 4.1
70	Beam dump	15	A	60.3 ± 0.2	36.8 ± 4.8
70	Beam dump	23	A	58.8 ± 0.2	40.1 ± 1.4
70	Phantom	NoRS	C	50.7 ± 0.9	11.2 ± 2.2
70	Phantom	10	C	29.1 ± 0.8	14.8 ± 3.8
70	Phantom	15	C	15.6 ± 0.7	24.9 ± 5.0
70	Phantom	23	C	22.0 ± 0.7	28.4 ± 4.9
70	Beam dump	NoRS	C	142.9 ± 1.6	13.7 ± 1.0
70	Beam dump	10	C	63.2 ± 0.2	29.4 ± 3.1
70	Beam dump	15	C	49.2 ± 0.2	35.9 ± 4.9
70	Beam dump	23	C	46.7 ± 0.2	35.0 ± 6.3
146	Phantom	NoRS	A	318.2 ± 0.5	35.1 ± 2.7
146	Phantom	10	A	1268 ± 2.0	16.2 ± 1.0
146	Phantom	15	A	2539 ± 4.0	11.7 ± 0.7
146	Phantom	23	A	2844 ± 4.0	10.4 ± 0.7
146	Beam dump	NoRS	A	496.0 ± 0.7	24.8 ± 1.5
146	Beam dump	10	A	2524.0 ± 3.7	10.7 ± 0.6
146	Beam dump	15	A	2678.3 ± 3.9	10.8 ± 0.6
146	Beam dump	23	A	2920.8 ± 4.3	10.5 ± 0.5
146	Phantom	NoRS	C	157.2 ± 1.8	21.8 ± 2.9
146	Phantom	10	C	128.6 ± 1.5	33.9 ± 2.4
146	Phantom	15	C	104.3 ± 1.3	40.1 ± 5.0
143	Phantom	23	C	116.5 ± 1.4	35.2 ± 4.1
146	Beam dump	NoRS	C	404.1 ± 4.3	23.4 ± 1.6
146	Beam dump	10	C	1448 ± 15	12.9 ± 0.9
146	Beam dump	15	C	1543 ± 16	12.1 ± 0.6
146	Beam dump	23	C	1723 ± 18	10.3 ± 0.8

Either the phantom or a proton beam dump was irradiated, and irradiations were made either with a range shifter at a certain range-shifter position (AG) or without the range shifter (NoRS). The values are given with two significant figures, and as mentioned in Section 2.4, the values are illustrated with statistical uncertainties only with k = 1. Effects from, e.g., uncertainties in gas pressure, detector dimensions, and the *W* value, give an additional contribution of 4%.

**Table 2:** Estimated *H* ^*^ values for different irradiation settings.

Proton energy (MeV)	Irradiated target	AG (cm)	Position	H^*^ (μSv/h)
70	Phantom	NoRS	A	377 ± 43
70	Phantom	10	A	372 ± 36
70	Phantom	15	A	442 ± 42
70	Phantom	23	A	426 ± 41
70	Beam dump	NoRS	A	586 ± 33
70	Beam dump	10	A	418 ± 42
70	Beam dump	15	A	421 ± 50
70	Beam dump	23	A	452 ± 44
146	Phantom	NoRS	A	2130 ± 140
146	Phantom	10	A	4420 ± 210
146	Phantom	15	A	6880 ± 310
146	Phantom	23	A	7120 ± 320
146	Beam dump	NoRS	A	2550 ± 130
146	Beam dump	10	A	6430 ± 240
146	Beam dump	15	A	6870 ± 270
146	Beam dump	23	A	7350 ± 250

Either the phantom or a proton beam dump was irradiated, and irradiations were made either with a range shifter at a certain range-shifter position (AG), or without the range shifter (NoRS). The values are given with two significant figures and as mentioned in Section 2.4, the values are illustrated with statistical uncertainties only with k = 1. Effects from, e.g., uncertainties in gas pressure, detector dimensions, and the *W* value give an additional contribution of 4%.

### 3.1 Measured absorbed dose as function of range-shifter position

**Figure 4** shows the absorbed dose rate in positions A and C as a function of range-shifter position, both when irradiating a phantom (**Figures 4A, B**) and when irradiating the proton beam dump by the far wall (**Figures 4C, D**). The dotted lines indicate the absorbed dose rate without a range shifter.

In the stray radiation field from the 70-MeV proton beam (**Figures 4A, C**), the absorbed dose decreased when applying a range shifter in both positions A and C, both with and without the presence of the phantom.

It is apparent that when the phantom was irradiated with a 146-MeV proton field (**Figure 4B**), the absorbed dose rate was dramatically increased in the A position when the range shifter was applied. The absorbed dose rate increased with the air gap. It is also notable that the dose rate when irradiated with a 146-MeV proton beam seemed to be independent of the presence of a phantom (**Figure 4B** *vs*. **4D**) at 15- and 23-cm range-shifter positions, which is an indication that the majority of the dose contribution came from the range shifter. In the C position, which was shadowed by the phantom when it was present, the dose rate decreased when the range shifter was applied. When irradiating without a range shifter and phantom, directly on the proton beam dump, it is noteworthy that the scattering from the range shifter was still higher than scattering from the beam dump.

### 3.2 Measured dose-mean lineal energy as a function of the range-shifter position

The covariance corrections in all measurements were small, confirming the stability of both the beam and the measurement system. This stability makes comparisons between ${\bar{y}}_{D}$ values at the different range-shifter positions more reliable.

**Figure 5** illustrates the ${\bar{y}}_{D}$ values for different range-shifter settings, when both the phantom (**Figures 5A, B**) and the proton beam dump (**Figures 5C, D**) were irradiated. When the phantom was irradiated with 70-MeV protons, the ${\bar{y}}_{D}$ values increased in both positions when a range shifter was inserted. In the A position, the ${\bar{y}}_{D}$ value seemed to be independent of the range-shifter position, while there was an increasing trend in the C position. The increasing ${\bar{y}}_{D}$ value indicates an increasing dose contribution from a high-LET component.

The most prominent results are seen in the right figures. When irradiating the phantom with a 146-MeV proton beam (**Figure 5B**), the ${\bar{y}}_{D}$ value measured in the A position decreased from approximately 35 to 15 keV/μm when applying the range shifter at a 10-cm air gap and then continued to decrease slightly with the increased air gap. The C side, being more shielded by the phantom, experienced the opposite—the ${\bar{y}}_{D}$ value increased, but there was no significant air gap dependence. Compared to the 70-MeV proton irradiation (**Figure 5A**), the ${\bar{y}}_{D}$ value at the C position increased, which can be explained by production of neutrons that were more highly energetic when irradiating with a 146-MeV proton beam than with a 70-MeV proton beam.

**Figure 5D** shows that when there was no phantom present, the ${\bar{y}}_{D}$ value in both positions decreased, indicating a larger contribution of a low-LET component from the range shifter when irradiating with a 146-MeV proton beam.

### 3.3 Simulated relative dose distributions

**Figure 6:**
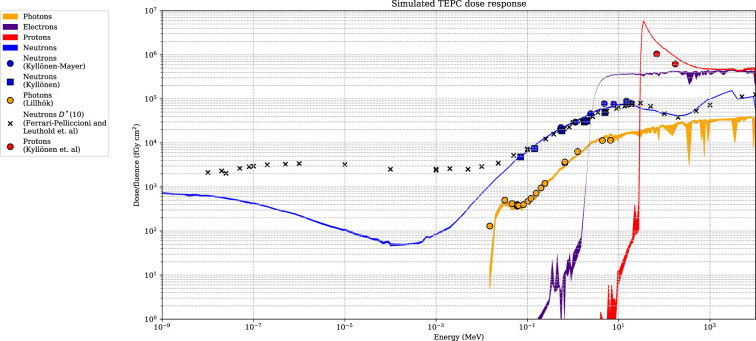
The dose-simulated response for the Sievert detectors, with associated uncertainties (k = 1) represented as colour bands. The simulated response is compared with experimental data from Kyllönen and Mayer (28), Kyllönen et al. (19), Lillhök (18), and Kyllönen et al. (21). Simulated conversion coefficients to ambient absorbed dose from Ferrari and Pelliccioni (29) and Leuthold et al. (30) are also included.

**Figure 7:**
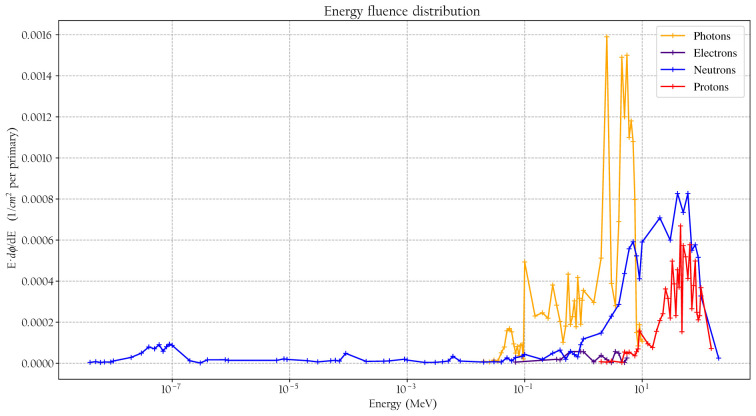
The simulated fluence distribution at position A when the phantom is irradiated with 146-MeV protons and a range shifter is applied with a 23-cm air gap.

**Figure 8:**
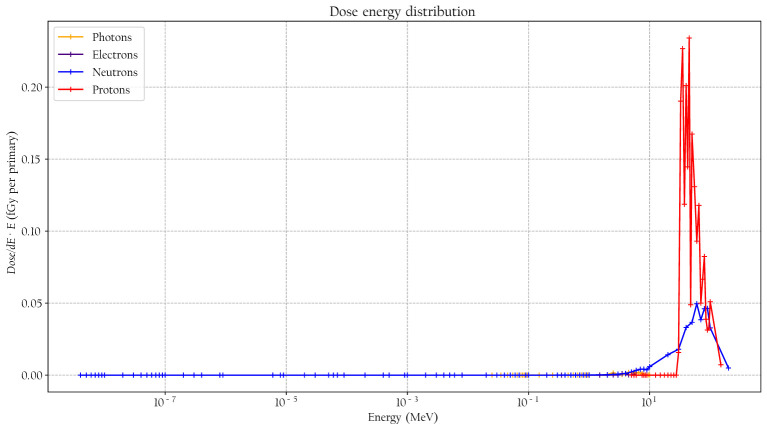
The simulated dose distribution at position A when the phantom is irradiated with 146-MeV protons and a range shifter is applied with a 23-cm air gap.

The simulated detector absorbed dose responses for neutrons, protons, photons, and electrons are presented in **Figure 6**. Included are also measured values, using the same detectors, for neutrons, photons, and protons from Lillhök (18), Kyllönen et al. (19, 21), and Kyllönen and Mayer (28), as well as conversion coefficients to ambient absorbed doses from Ferrari and Pelliccioni (29) and Leuthold et al. (30). The conversion coefficients from Ferrari and Pelliccioni were calculated as the ratio of the reported *H* ^*^ conversion coefficient and the effective quality factor at 10-mm depth in the ICRU sphere. A good agreement is seen between the simulated absorbed dose response and previously published experimental values for proton, neutrons, and photons. It can also be noted that the simulated detector absorbed dose and the ambient absorbed dose agree well for high neutron energies above 20 MeV where no experimental data were available. The energy-weighted fluence distributions at position A when the phantom is irradiated with a 146-MeV proton beam and the range shifter is applied at a 23-cm air gap are illustrated in **Figure 7**. The dose energy distribution for the same irradiation settings and position in **Figure 8** shows that the dose deposited in the detector is mainly from > 10-MeV protons.

**Figure 9:**
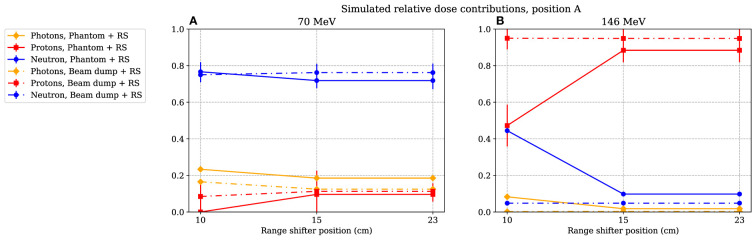
The simulated relative dose contribution at position A when the phantom or proton beam dump was irradiated with 70- and 146-MeV protons **(A)** and **(B)** respectively. The range-shifter positions correspond to the air gaps when the phantom was present. The lines between data points are used as guide for the eye. The dashed–dotted lines show the simulated relative dose contribution when no phantom was present, while the solid lines show the contributions when the phantom was irradiated. The values are illustrated with one standard uncertainty.

In **Figure 9**, the simulated relative dose distributions from photons, neutrons, and protons are shown for the A position. When irradiating with a 70-MeV proton beam and using a range shifter, the majority of the dose is due to neutrons, as indicated by the increase in ${\bar{y}}_{D}$ value seen in **Figure 5A**. During 146-MeV proton beam irradiations, the neutron contribution in position A decreases with increasing range-shifter position while the proton contribution increases sharply. At 15 and 23 cm, the relative contributions from the radiation components does not change significantly. We note here that also the ${\bar{y}}_{D}$ value was relatively constant when comparing the 15- and 23-cm range-shifter positions (see **Figure 5B**).

The simulations were performed with a simple geometry, only including the detector, range shifter, phantom, and proton beam dump. To investigate the scattering effect of the surrounding structure, more thorough and hence time-consuming simulations were performed for a few cases, using a more comprehensive geometry modelled by Ardenfors et al. (27). These simulations showed that the surrounding walls were important when relatively lowly absorbed doses were measured, particularly in position C, where the range shifter was shielded by the phantom. These complementary simulations did not change the conclusion that the primary beam was scattered in the range shifter. In position A, the increased absorbed dose was still completely dominated by the relative contribution of the scattered protons deposited in the detector.

From the simulated dose distribution, it is also evident that the contribution from photons is very low (see **Figure 9**), so any low-LET components leading to a decrease in the resulting ${\bar{y}}_{D}$ value at 146 MeV were likely to come from protons.

### 3.4 Estimated dose equivalent as a function of the range-shifter position

**Figure 10:**
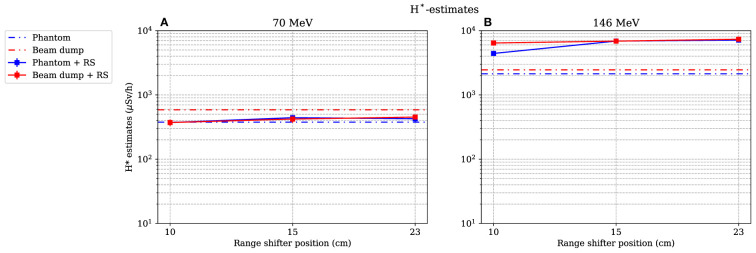
Estimation of the dose equivalent at position A, when both the phantom and the proton beam dump are irradiated with 70- and 146-MeV protons **(A)** and **(B)** respectively. The lines between data points are used as guide for the eye. The dashed–dotted lines show the levels without a range shifter, i.e., scattered from the phantom or from the beam dump. As mentioned in Section 2.4, the values are illustrated with statistical uncertainties with k = 1.

As for the simulated dose contributions, only the investigation of position A is presented here. The dose equivalents as functions of different range-shifter settings were estimated during irradiations of both the phantom and the proton beam dump (see **Figure 10**). In **Figure 10A**, for the 70-MeV proton beam, it is seen that the *H* ^*^ values were relatively constant even after applying a range shifter. The absorbed dose rate was lower, and the increase of high-LET neutrons was not high enough to give a higher *H* ^*^ value. During irradiation with a 146-MeV proton beam, two noteworthy results can be pointed out. First, the *H* ^*^ value without a range shifter was higher than when irradiating with a 70-MeV proton beam, indicating that the stray field from the phantom alone led to an increase with an increased proton beam energy. This increase is expected since the proton energy was more than double. Second, when applying the range shifter, the *H* ^*^ values increased by almost a factor of 2 (for the smallest range-shifter position when irradiating the phantom), up to a factor more than 3 (23-cm air gap). At the 10-cm air gap, position A was partly shadowed by the phantom, which explains the air gap dependence. The increase in the *H* ^*^ value here reflects the significantly higher absorbed doses by the scattered protons.

## 4 Discussion

**Figure 11:**
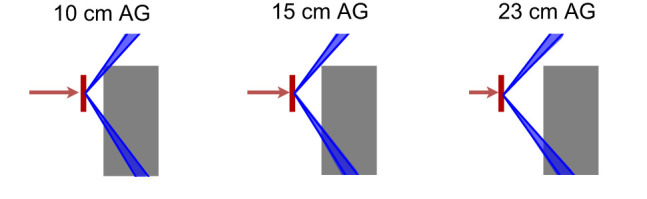
The blue cones indicates the path from the centre of the range shifter (red) to the borders of the detectors at positions A (top) and C (bottom). When the air gap is small, a particle that travels in a straight line needs to travel through more phantom material (grey) than when the air gap is large. This can be one reason to the air gap dependency that is seen for position C in **Figure 5A**.

When irradiating the phantom with 70-MeV protons, the absorbed dose rate at both positions A and C decreased and the ${\bar{y}}_{D}$ value increased when applying the range shifter, indicating a larger contribution from a high-LET component. In position C, an air gap dependency which was not seen in position A was noticed. The detected neutrons in position A were less moderated by the phantom even at small air gaps, while the neutrons that reached position C had a larger moderation due to more phantom material to penetrate. At larger air gaps, the exposed phantom material was reduced and the neutrons deposited were therefore more energetic. This is visualised in **Figure 11**, where the blue cones indicate the line of sight from the centre of the range shifter to the detectors at positions A and C. At larger air gaps, a particle travelling in a straight line from the range shifter to a detector passes through less phantom material than at smaller air gaps.

When the phantom was irradiated with 146-MeV protons, the absorbed dose rate in position A was significantly increased, which can be explained by protons scattering at a large enough angle from the range shifter to miss the phantom. A clear dependency on the air gap was observed. When the air gap was small, the phantom shadowed the detector, while at larger air gaps, the detector saw almost no effect from the phantom. The hypothesis that high-energy (>10-MeV) protons represent the main part of the dose contribution in the detector at position A is supported by the measured decrease in the ${\bar{y}}_{D}$ value when the range shifter is applied, indicating a large contribution from a low-LET component. The simulated dose contributions agreed with the measurements and also indicated that contribution from photons was low, which further supports the hypothesis that the majority of the low-LET component comes from protons.

The *H* ^*^ value is an approximation of the ambient dose equivalent, *H* ^*^(10), and was calculated using a linear approximation. The differences between the ICRP Q value and this approximation is a source of uncertainty. In addition, e.g., back-scattering from the tissue material in the ICRU sphere was not detected in gas-filled TEPCs such as the Sievert detectors. Therefore, the absorbed dose from neutrons at intermediate energies is underestimated. An uncertainty of around 15%–25% has been estimated due to the abovementioned effects by Lillhök (18). The estimated *H* ^*^ value during irradiations with 146-MeV protons was however several times larger with a range shifter than without and clearly exceeded the uncertainties in the ambient dose equivalent estimation even if the neutron energy distribution would change.

It is well established that the stray field in proton therapy contains both thermal and high-energy *neutron* components and that the magnitude of the contributions depends strongly on positions and proton beam energies (2, 4, 5). However, when applying a range shifter, the contributions at some positions that are less shielded by the phantom are not necessarily dominated by neutrons, and the thermal neutron contribution can be suppressed, as illustrated in **Figures 7**, **8**. The range shifter can dramatically affect the stray radiation field. The potential presence of protons needs to be considered and included in simulations and measurements.

## 5 Conclusion

In a previous campaign, measuring the out-of-field neutron doses in a proton pencil beam facility, a significant increase in absorbed dose measured by a TEPC was detected at one position when a range shifter was applied. The measurements in the present study reproduced this increase, and measurements of the dose-mean lineal energy confirmed that the increased dose rate during irradiation with a 146-MeV proton beam consisted of low-LET radiation. The results were supported by Monte Carlo simulations showing that the low-LET component consisted of high-energy (>10-MeV) protons. The greatly enhanced dose rate when using the range shifter resulted in an up to three times higher dose equivalent compared to when no range shifter was applied. The results illustrate the importance of considering the potential dose contribution from protons in out-of-field simulations as well as using instruments sensitive also to proton radiation during measurement campaigns.

## Data availability statement

The raw data supporting the conclusions of this article will be made available by the authors, without undue reservation.

## Author contributions

LE, JL, and TB planned the experimental measurements. RB-M performed the simulations. AD and ML planned and performed the irradiations at the Skandion Clinic. LE and JL performed pre-calibrations and preparations of the instruments. LE, JL, AD, and ML performed the measurements. LE performed the data analysis. LE did the main part of the writing and made the figures. All authors contributed to the article and approved the submitted version.

## Funding

This project was funded by the Swedish Radiation Safety Authority, grant agreement no. 7030265-00. In addition, funding was received from Euratom’s research and innovation programme 2019-20 under grant agreement no. 945196.

## Acknowledgments

The authors would like to thank Oscar Ardenfors for providing a simulation geometry model for the Skandion treatment room. The Swedish National Metrology Laboratory for Ionising Radiation provided radioactive sources and equipment for control measurements and are gratefully acknowledged.

## Conflict of interest

The authors declare that the research was conducted in the absence of any commercial or financial relationships that could be construed as a potential conflict of interest.

## Publisher’s note

All claims expressed in this article are solely those of the authors and do not necessarily represent those of their affiliated organizations, or those of the publisher, the editors and the reviewers. Any product that may be evaluated in this article, or claim that may be made by its manufacturer, is not guaranteed or endorsed by the publisher.

## Footnotes

1
https://skandionkliniken.se/en/
